# Surgical outcomes of laparoscopic proximal gastrectomy for upper-third gastric cancer: esophagogastrostomy, gastric tube reconstruction, and double-tract reconstruction

**DOI:** 10.1186/s12893-023-02219-9

**Published:** 2023-10-12

**Authors:** Jianhua Chen, Fei Wang, Shuyang Gao, Yapeng Yang, Ziming Zhao, Jiahao Shi, Liuhua Wang, Jun Ren

**Affiliations:** 1 Department of Clinical Medical College, The Yangzhou School of Clinical Medicine, Dalian Medical University, Yangzhou, People’s Republic of China; 2 Department of General Surgery, Northern Jiangsu People’s Hospital, Clinical Medical School, Yangzhou University, 98 Nantong West Road, Yangzhou, 225001 People’s Republic of China; 3 Yangzhou Key Laboratory of Basic and Clinical Transformation of Digestive and Metabolic Diseases, Yangzhou, People’s Republic of China; 4 Department of General Surgery, General Surgery Institute of Yangzhou, Northern Jiangsu People’s Hospital, Yangzhou, People’s Republic of China

**Keywords:** Gastric cancer, Laparoscopic proximal gastrectomy, Esophagogastrostomy, Double-tract reconstruction, Gastric tube reconstruction

## Abstract

**Background:**

There is no consensus on the optimal reconstruction technique after proximal gastrectomy. The purpose of this study was to retrospectively compare the surgical outcomes among esophagogastrostomy (EG) anastomosis, gastric tube (GT) reconstruction and double-tract (DT) reconstruction in patients who underwent laparoscopic proximal gastrectomy (LPG) to clarify the superior reconstruction method.

**Methods:**

This study enrolled 164 patients who underwent LPG at the Northern Jiangsu People's Hospital in Jiangsu between January 2017 to January 2022 (EG: 51 patients; GT: 77 patients; DT: 36 patients). We compared the clinical and pathological characteristics, surgical features, postoperative complications, nutritional status, and quality of life (QOL) among the above three groups.

**Results:**

Mean operative time was longer with the DT group than the remaining two groups (*p* = 0.001). With regard to postoperative complications, considerable differences in the postoperative reflux symptoms (*p* = 0.042) and reflux esophagitis (*p* = 0.040) among the three groups were found. For the nutritional status, total protein, hemoglobin and albumin reduction rates in the GT group were significantly higher than the other two groups at 12 months postoperatively. In the PGSAS-45, three assessment items were better in the DT group significantly compared with the esophageal reflux subscale (*p* = 0.047, Cohen’s d = 0.44), dissatisfaction at the meal (*p* = 0.009, Cohen’s d = 0.58), and dissatisfaction for daily life subscale (*p* = 0.012, Cohen’s d = 0.56).

**Conclusions:**

DT after LPG is a valuable reconstruction technique with satisfactory surgical outcomes, especially regarding reduced reflux symptoms, improving the postoperative nutritional status and QOL.

## Introduction

Gastric cancer (GC) has the fifth highest incidence of all cancers globally, what is even more frightening is that GC is also the fourth leading cause of cancer-related death and mortality in the word [1]. Proximal gastric cancer (PGC), referred to as upper-third stomach cancer, has been on the rise recently [2, 3]. Early-stage PGC cases have also increased [4]. Surgery remains the cornerstone of treatment for PGC, which includes total gastrectomy (TG) and proximal gastrectomy (PG). Following the Japanese Gastric Cancer Guidelines (JGCG) [5], TG or distal gastrectomy (DG) is the recommended surgical treatment for clinically node-positive (cN +) or T2-T4a tumors. PG is recommended as a function-preserving surgical method only for cT1N0 GC in PGC.

The relevant literature reported that patients who undergo gastrectomy for stomach cancer always experience postoperative body weight loss, and the considerable loss of body weight and subsequent sarcopenia are detrimental to long-term survival [6, 7]. Therefore, the maintenance of body weight and nutritional condition following a gastrectomy must receive careful consideration. TG can lead to postoperative malnutrition even if it guarantees greater tumour margins and a more thorough lymphadenectomy. Compared with TG, PG preserves a portion of the stomach, facilitating weight maintenance and enhancing the postoperative nutritional condition and quality of life (QOL) [8, 9]. However, most surgeons tend to choose TG, even for early PGC. The reason for this phenomenon is the high incidence of complications after simple esophagogastrectomy, especially reflux esophagitis, which can causes severe heartburn, chest pain, acid reflux and anorexia and dramatically affects the patient's postoperative QOL [10]. A few reconstruction techniques have been devised after PG to overcome this difficulty. The JGCG recommends three different reconstruction options for PG, including esophagogastrostomy (EG) anastomosis, double-tract (DT) method, and jejunal interposition (JIP) [5]. Furthermore, jejunal pouch interposition (JPI) and gastric tube (GT) reconstruction are also considered viable techniques. Among them, GT is a modified anti-reflux reconstruction based on EG, which reduce the severity of reflux symptoms by cutting a tube to extend the distance of the reflux and reduces the gastric acid secretion. However, there is no consensus on the optimal reconstruction technique after PG. The purpose of this study was to retrospectively compare and analysis the surgical outcomes among the EG, GT, and DT reconstruction in patients who underwent LPG to clarify the superior reconstruction method.

## Materials and methods

### Patients

From January 2017 to January 2022, we retrospectively enrolled 164 patients with strict criteria who were diagnosed with PGC and underwent PG at the Northern Jiangsu People's Hospital in Jiangsu, China. All patients underwent gastrectomy by laparoscopic approach. Fifty-one patients underwent direct anastomosis of the esophagus to the residual stomach after PG and were enrolled in the EG group. Seventy-seven patients who underwent PG had GT reconstruction and were categorized as the GT group. Thirty-six patients treated with the double tract method after PG were denoted as the DT group. Our inclusion criteria were (1) patients with PGC underwent PG, and more than one-half of the remnant stomach was preserved; (2) intraoperative and postoperative pathology confirmed the tumor was located in the superior third of the stomach, and pathology reports suggested negative cut margins; (3) survival time of more than one year after surgery; (4) preoperative gastroscopy confirmed no ulcers, polyps or tumours in the gastric sinus or duodenal bulb; (5) no neoadjuvant chemotherapy was received before surgery; (6) patients was fully capable of understanding and answering questionnaires; (7) the patient had no other diseases or surgical history that would interfere with the response. Exclusion criteria: (1) patients with preoperative combined gastrectomy for other malignant tumours or benign diseases; (2) patients with preoperative combined severe anaemia (hemoglobin < 70 g/L), hypoproteinemia (albumin < 30 g/L) and coagulation dysfunction (platelet < 50*10^9^/L) requiring preoperative intervention; (3) patients with preoperative combined severe comorbidities, such as liver cirrhosis, chronic renal failure, myocardial infarction or respiratory diseases; (4) patients with preoperative combined disorders of consciousness and mental system diseases; (5) patients with other malignant tumors or with other organs resected at the same time due to other diseases.

Before 2019, EG was the first choice for reconstruction in PG. From 2019 to April 2020, GT and DT were used for reconstruction after PG. When patients were found to be ineligible for esophagogastrostomy due to the required excessive stomach resection, the surgeons chose DT after PG. DT was the first choice of reconstruction after April 2020. The same surgery team performed all operation procedures, and all patients were managed with the same postoperative treatment when staying in the hospital. All patients with advanced gastric cancer were advised to receive standard postoperative chemotherapy with XELOX (capecitabine plus oxaliplatin).

We recorded information (reconstruction method and surgery date) on patients who underwent PG and did not make any interventions or notifications between discharge and the 1-year postoperative review. If the patients met the inclusion criteria, we informed them about all the study matters at the 1-year postoperative review. It was up to the patients to decide whether or not to be enrolled in the study. Based on the Declaration of Helsinki, each patient has agreed and signed an informed consent form after careful consideration. The protocol was approved by the committee of the Northern Jiangsu People's Hospital (2019KY-022).

### Surgical procedures

#### Laparoscopic proximal gastrectomy

By using the open technique, a 12-mm port was placed via the inferior of the umbilicus, and 10 mmHg of carbon dioxide (CO_2_) was injected into the peritoneal cavity. The other four working ports were placed under laparoscopic guidance, consisting of a 12-mm port on the left mid-clavicular line at the umbilicus and a 5-mm port on the right mid-clavicular line 2–3 cm above the umbilicus, two 5-mm ports on the right mid-clavicular and left mid-axillary lines below the costal margin. According to the JGCG [5], PG was completed with D2 lymphadenectomy. The right gastroepiploic and right gastric arteries were preserved during surgery to maintain blood supply to the residual stomach. To retain pyloric function, the vagus nerve's hepatic branch was also intact. Reconstruction was performed after the frozen inspection verified that the resection margins were tumor-negative.

#### Reconstruction for PG with EG

The proximal esophagus was resected through an endoscopic linear stapler. The specimen was removed with a linear device closure at the line from the lower middle third of the lesser curvature to the junction of the right and left vascular arches of the greater curvature of the stomach. A small incision was made in the anterior wall of the remnant stomach. Reconstruction was performed by an end-to-side anastomosis with a circular stapler between the esophagus and the anterior wall of the remnant stomach, and no anti-reflux procedure was performed.

#### Reconstruction for PG with GT

The proximal esophagus was resected through an endoscopic linear stapler. After ligating the vessels of the greater and lesser curvature of the stomach, the gastric body was cut to make the gastric tube by using a linear anastomosis. The remnant stomach was cut into a tube 15 cm long and 3 cm wide. The esophageal stump was anastomosed on the anterior wall of the gastric tube through a circular anastomosis.

#### Reconstruction for PG with DT

The jejunal mesentery was incised 25 cm distal to the flexor ligament, the small intestinal mesenteric vessels were ligated, and the distal intestinal canal was freed for about 15 cm. The jejunum was incised at 25 cm from the ligament of Treitz using a linear stapler and closed the proximal jejunal stump. The distal intestine was lifted, and the esophagojejunostomy (E-Jstomy) was performed using a linear stapler. Next, in side-to-side gastrojejunostomy (G-Jstomy), one hole was made at the jejunum 15 cm caudal from the E-Jstomy and in the anterior wall of the remnant stomach 2 cm from the incision edge. Facing cephalad, a linear anastomosis was inserted into the gastric and jejunal anastomoses for anastomosis. A side-to-side jejunojejunostomy (J-Jstomy) was made by an anastomosis between the anal-side jejunum and oral-side jejunum at 35 cm from the stump.

Stomach remnants and anastomosis were reinforced with sutures to prevent leakage. The same surgery team performed all operation procedures, and all patients were managed with the same postoperative treatment when staying in the hospital.

#### Data collection and assessment

All patients were follow-up in the outpatient clinic during the first, sixth, and twelfth months after discharge. We collected patients' medical and follow-up records to facilitate statistics and analysis of data. This study provides a retrospective analysis of the three reconstruction methods through the following aspects: clinical and pathological characteristics; surgical outcomes; postoperative complications; nutritional status; QOL Assessment. Clinical and pathological characteristics include age; sex; American Society of Anesthesiologists (ASA) Classification; Body Mass Index (BMI); tumour size; histological type; differentiated degree; total lymph nodes and positive lymph nodes; pathologic T, N, and M stage (the Japanese Classification of Gastric Carcinoma, 3rd English edition); adjuvant chemotherapy. Surgical outcomes include blood loss; operative time; postoperative hospital stays, and time of first postoperative liquid diet. Postoperative complications were categorized and recorded according to the Clavien–Dindo classification [11], and the complication classification higher than Clavien–Dindo classification IIIa were defined as major complications.

We used gastrointestinal fiberscopes to assess the incidence and severity of reflux esophagitis, and the Los Angeles (LA) classification system [12] to classify reflux esophagitis. The total protein (TP), serum albumin (ALB) and hemoglobin (HB) levels at the first, sixth, and twelfth months after surgery and the body weight changes one year after surgery were collected. The preoperative indexes were used as the baseline to analyze the postoperative nutritional conditions of patients.

The Postgastrectomy Syndrome Assessment Scale-45 (PGSAS-45) [13] was created to accurately assess the symptoms, daily living status, and QOL in the undergoing gastrectomy patients. When patients were reviewed postoperatively (≥ one year), patients received and completed this scale. We summarized and counted all the results of the questionnaire. Among them, we categorized the twenty-three items into seven subscales and included 12 items as primary outcomes to analyze and compare the QOL among the three groups. The subscale scores reflected the mean scores for the component items, whereas the total symptom score was based on the mean of the seven subscale scores. The twenty-three symptom items were scored using the seven-grade Likert scale. Other were scored using the five-grade Likert scale. Higher scores were considered to have better conditions in 1–8, 34, 35 and 38–40. On the contrary, higher scores were regarded as worse conditions in other items [13].

#### Statistics

All statistical calculations were conducted using the SPSS Statistics ver. 27.0 software. Fisher’s exact test and the analysis of variance (ANOVA) were used to compare the data among the three groups. A *P*-value of less than 0.05 was considered statistically significant. In addition, QOL Assessments were further analyzed using multiple comparison method. In case the *P* value of ANOVA was less than 0.1, Tukey was conducted. When the *P* values were less than 0.1 in Tukey, Cohen’s d was performed for the purpose of effect size [14, 15]. Cohen's d refers to the effect of individual-caused variables: the effect size from 0.2 to 0.5 indicates a small difference clinically; from 0.5 to 0.8 demonstrates a moderate effect; and more than 0.8 denotes a large effect clinically.

## Results

### Clinical and pathological characteristics

One hundred sixty-four patients were enrolled in this study (EG: 51 patients; GT: 77 patients; DT: 36 patients). The patient and tumor characteristics are summarized in Table 1. All groups had a majority of males. There were 36 (70.6%) males and 15 (29.4%) females with an average age of 69.10 ± 6.98 years in the EG group. In the GT group, there were 50 (71.4%) males and 22 (28.6%) females; the average age was 68.53 ± 7.29 years. The DT group included 23 (63.9%) males and 13 (36.1%) females with an average age of 68.81 ± 6.33 years. No significant differences in sex, age, BMI, ASA-PS, tumor size, histological type, differentiated degree, total lymph nodes, positive lymph nodes, pathological T stage, pathological stage and adjuvant chemotherapy were noted among the groups (Table 1).

**Table 1:** Clinical and pathological characteristics

**Variables**	**EG** ***n* = 51**	**GT** ***n* = 77**	**DT** ***n* = 36**	***p* value**
Gender				0.705
Male	36(70.6%)	55(71.4%)	23(63.9%)	
Female	15(29.4%)	22(28.6%)	13(36.1%)	
Age (years)	69.10 ± 6.98	68.53 ± 7.29	68.81 ± 6.33	0.904
BMI (kg/m^2^)	24.29 ± 3.67	23.76 ± 2.68	23.25 ± 2.64	0.281
ASA-PS				0.056
I	1(2.0%)	2(2.6%)	1(2.8%)	
II	31(60.8%)	54(70.1%)	32(88.9%)	
III	19(37.2%)	21(27.3%)	3(8.3%)	
Tumor size (cm)	3.36 ± 0.99	3.69 ± 0.99	3.65 ± 1.01	0.171
Histological type				0.564
Adenocarcinoma	45(88.2%)	71(92.2%)	34(94.4%)	
Other	6(11.8%)	6(7.8%)	2(5.6%)	
Differentiated degree				0.765
Well /Moderately differentiated	18(35.3%)	31(40.3%)	12(33.3%)	
Poorly differentiated	27(52.9%)	40(51.9%)	22(61.1%)	
Other	6(11.8%)	6(7.8%)	2(5.6%)	
Total lymph nodes	15.02 ± 5.47	15.68 ± 7.45	15.36 ± 9.00	0.883
Positive lymph nodes	0.24 ± 0.59	0.26 ± 0.66	0.19 ± 0.53	0.868
Pathological T stage ^a^				0.104
T1	27(52.9%)	44(57.1%)	25(69.4%)	
T2	8(15.7%)	22(28.6%)	5(13.9%)	
T3	3(5.9%)	2(2.6%)	0(0.0%)	
T4	13(25.5%)	9(11.7%)	6(16.7%)	
Pathological N stage ^a^				0.806
N0	43(84.2%)	66(85.7%)	31(86.1%)	
N1	6(11.8%)	5(6.5%)	3(8.3%)	
N2	1(2.0%)	5(6.5%)	2(5.6%)	
N3	1(2.0%)	1(1.3%)	0(0.0%)	
Pathological Stage ^a^				0.101
I	30(58.8%)	60(77.9%)	28(77.8%)	
II	10(19.6%)	9(11.7%)	2(5.6%)	
III	11(21.6%)	8(10.4%)	6(16.7%)	
Adjuvant chemotherapy	16(31.4%)	13(16.9%)	6(16.7%)	0.109

*BMI* Body Mass Index, *ASA-PS* American Society of Anesthesiologists physical status classification system, Variables are described using mean ± standard deviations

*p < 0.05, significant differences among three groups

^a^According to the Japanese Classification of Gastric Carcinoma, 3rd English edition

### Surgical outcomes

All patients underwent gastrectomy by laparoscopic approach. Operative outcomes are summarized in Table 2. The mean operative time was remarkably longer with the DT group than the remaining two groups (*p* = 0.001). The mean blood loss was less in the EG group than in the remaining two groups, but the difference was insignificant (*p* = 0.594). No significant differences in the mean time of the first postoperative liquid diet was noted among the groups.

**Table 2:** Surgical outcomes and postoperative complications

Variables	EG *n* = 51	GT *n* = 77	DT *n* = 36	*p* value
Blood loss(ml)	65.49 ± 30.68	73.51 ± 58.28	72.78 ± 26.58	0.594
Operative time(min) *	138.53 ± 28.15	141.38 ± 33.54	163.06 ± 36.63	0.001*
Postoperative hospital stay(days)	11.39 ± 2.15	10.83 ± 2.20	11.72 ± 2.20	0.101
Time of first postoperative liquid diet(days)	6.80 ± 1.50	6.57 ± 1.70	6.64 ± 1.38	0.712
Postoperative complications	23(45.1%)	25(32.5%)	8(22.2%)	0.078
Major complications(C-D ^a^ ≥ IIIa)				
Anastomotic bleeding	0(0.0%)	1(1.3%)	0(0.0%)	0.566
Anastomotic stenosis	1(2.0%)	4(5.2%)	2(5.6%)	0.615
Minor complications(C-D ^a^ < IIIa)				
Anastomotic leakage	2(3.9%)	0(0.0%)	0(0.00%)	0.106
Intestinal obstruction	2(3.9%)	2(2.6%)	1(2.8%)	0.908
Pulmonary infection	1(2.0%)	1(1.3%)	0(0.0%)	0.711
Delayed gastric emptying	7(13.7%)	7(9.1%)	1(2.8%)	0.218
Symptom of reflux*	23(45.1%)	30(39.0%)	7(19.4%)	0.042*
Reflux esophagitis*	19(37.3%)	18(23.4%)	5(13.9%)	0.040*
Los Angeles classification				0.160
A	11(21.6%)	6(7.8%)	2(5.6%)	
B	5(9.8%)	7(9.1%)	2(5.6%)	
C	3(5.9%)	5(6.5%)	1(2.8%)	
D	0(0.0%)	0(0.0%)	0(0.0%)	

Variables are described using mean ± standard deviations

*p < 0.05, significant differences among three groups

^a^According to the Clavien-Dindo classification

### Postoperative complications

There was no mortality or recurrence among the three groups in the twelve-month follow-up. 23 (45.1%) patients versus 25 (32.5%) patients versus 8 (22.2%) patients (*p* = 0.078) were diagnosed with postoperative complications in the EG, GT, and DT group, respectively. Anastomotic stenosis and anastomotic bleeding were classified as major complications. In the GT group, one patient was diagnosed with anastomotic bleeding and treatment by endoscopic hemostasis. One (2.0%) patient in the EG group, four (5.2%) patients in the GT group, and 2 (5.6%) patients in the DT group showed anastomotic stenosis and needed dilatation by balloon dilatation under endoscopy. Minor complications included anastomotic leakage, pulmonary infection, delayed gastric emptying, intestinal obstruction and reflux esophagitis. Two (3.9%) patients showed anastomotic leakage in the EG group. Incidences of intestinal obstruction for EG, GT, and DT group were 3.9% (two patients), 2.6% (two patients), and 2.8% (one patient), respectively. Patients diagnosed with anastomotic leakage and intestinal obstruction have all been successfully managed with conservative medical treatment. Pulmonary infection and delayed gastric emptying were not meaningful differences among the three groups (2.0% vs 1.3% vs 0.0%, respectively; *p* = 0.711; and 13.7% vs 9.1% vs 2.8%, respectively; *p* = 0.908).

Significant differences in the postoperative reflux symptoms (*p* = 0.042) and reflux esophagitis (*p* = 0.040) among the three groups were found in the twelve-month follow-up. Accumulatively, 23 (45.1%) patients in the EG group reported reflux symptoms, and 19 (37.3%) patients were diagnosed to have reflux esophagitis. There were 30 (39.0%) patients with reflux symptoms and 18 (23.4%) patients with reflux esophagitis in the GT group. Seven (19.4%) patients had reflux symptoms in the DT group, and five (13.9%) patients had reflux esophagitis by endoscopy. However, we observed no group-dependent differences in the Los Angeles Classification among the three groups (*p* = 0.160). In the EG group, 11 (21.6%) cases were classified as level A, 5 (9.8%) cases were classified as level B, and 3 (5.9%) cases were classified as level C. In the GT group, six (7.8%) patients were Grade A, seven (9.1%) patients were Grade B, and five (6.5%) patients were Grade C. In the DT group, 2 (5.6%) patients were Grade A, 2 (5.6%) patients were classified as level B, and one (2.8%) patient was classified as level C.

### Nutritional status

**Fig. 1:**
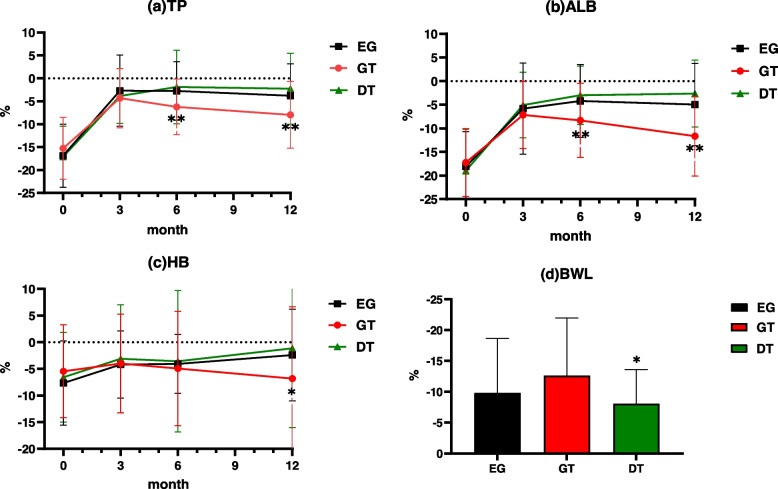
Comparison of nutritional outcomes in the (**a**) total protein (TP), (**b**) albumin (ALB), (**c**) hemoglobin (HB), and (**d**) body weight loss (BWL), among the three groups. All postoperative data are represented as percent reduction (mean ± SE) over preoperative data. *p < 0.05, **p < 0.01

Figure 1a-c shows the TP, ALB and HB levels in the three groups of patients one year after surgery. TP and ALB reduction rates did not differ significantly by the group during the three months after PG. However, the reduction rates in the GT group increased three months after PG. TP and ALB reduction rates in the GT group were markedly higher than the other two groups at 6 and 12 months postoperatively. In addition, HB reduction rates did not significantly differ among the three groups at 3 and 6 months postoperatively (*p* = 0.832, *p* = 0.711). Of clinical significance, HB reduction rates tended to be higher in the GT group than in the other two groups at 12 months postoperatively (-2.4% ± 8.6% vs -6.8% ± 13.5% vs -1.1% ± 14.9%, *p* = 0.039). The percentage of body weight loss (%BWL) at one year is shown in Fig. 1d. Mean ± SD %BWL at one year postoperatively was -9.8 ± 8.8% in the EG group, -12.6 ± 9.4% in the GT group, and -8.08 ± 5.5% in the DT group, with a significant difference among the three groups (*p* = 0.021).

### QOL Assessment

The nineteen preliminary outcome scales of PGSAS-45 were analyzed by ANOVA test (Table 3). Multiple comparisons (Tukey test) were performed on the esophagal reflux subscale, change in body weight, dissatisfaction at the meal and dissatisfaction for daily life subscale. The change in body weight was more in the GT group markedly compared with the EG group (*p* = 0.093, Cohen’s d = 0.31) and DT group (*p* = 0.002, Cohen’s d = 0.55). Three assessment items were better in the DT group compared with the GT group and EG group: the esophagal reflux subscale, dissatisfaction at the meal and dissatisfaction for daily life subscale. The scores for the esophageal reflux subscale (*p* = 0.047, Cohen’s d = 0.44), dissatisfaction at the meal (*p* = 0.009, Cohen’s d = 0.58), and dissatisfaction for daily life subscale (*p* = 0.012, Cohen’s d = 0.56) were less in the DT group than the EG group. The scores for the esophageal reflux subscale (*p* = 0.046, Cohen’s d = 0.41), dissatisfaction at the meal (*p* = 0.051, Cohen’s d = 0.40), and dissatisfaction for daily life subscale (*p* = 0.064, Cohen’s d = 0.38) were less in the DT group than the GT group. No meaningful differences were observed in the outcomes of the other assessment items.

**Table 3:** Seven subscales and twelve primary outcomes of PGSAS-45

Factor and item	EG *n* = 51	GT *n* = 77	DT *n* = 36	ANOVA *P* value	Tukey	*P* value	Cohen’s d
(Symptoms)							
Esophageal reflux subscale	2.73 ± 1.43	2.71 ± 1.52	2.10 ± 1.46	**0.088**	EG versus. GT	0.933	
					EG versus. DT	**0.047**	0.44
					GT versus. DT	**0.046**	0.41
Abdominal pain subscale	1.60 ± 0.59	1.68 ± 0.97	1.51 ± 0.63	0.565			
Meal-related distress subscale	2.00 ± 0.92	2.10 ± 1.01	2.06 ± 1.10	0.849			
Indigestion subscale	2.10 ± 0.64	1.95 ± 0.59	2.08 ± 0.69	0.346			
Diarrhea subscale	1.59 ± 0.90	1.39 ± 0.73	1.75 ± 1.26	0.144			
Constipation subscale	1.76 ± 1.27	1.58 ± 1.11	1.33 ± 0.59	0.185			
Dumping subscale	1.74 ± 0.92	1.85 ± 0.93	1.81 ± 0.92	0.810			
Total symptom score	1.93 ± 0.47	1.90 ± 0.54	1.81 ± 0.52	0.530			
(Living status)							
Change in body weight(%) *	-9.83 ± 8.85	-12.63 ± 9.36	-8.08 ± 5.53	**0.021**	EG versus. GT	**0.093**	0.31
					EG versus. DT	0.259	
					GT versus. DT	**0.002**	0.55
Ingested amount of food per meal*	6.20 ± 1.56	6.12 ± 1.49	6.44 ± 2.38	0.647			
Necessity for additional meals	2.08 ± 0.72	2.22 ± 0.91	2.11 ± 1.21	0.670			
Quality of ingestion subscale*	3.74 ± 0.56	3.60 ± 0.68	3.85 ± 0.73	0.140			
Ability for working	2.22 ± 0.70	2.39 ± 1.08	2.03 ± 0.65	0.126			
(QOL)							
Dissatisfaction with symptoms	2.45 ± 0.83	2.48 ± 1.05	2.11 ± 0.92	0.142			
Dissatisfaction at the meal	2.78 ± 0.86	2.64 ± 0.96	2.25 ± 1.00	**0.031**	EG versus. GT	0.374	
					EG versus. DT	**0.009**	0.58
					GT versus. DT	**0.051**	0.40
Dissatisfaction at working	2.06 ± 0.86	1.97 ± 0.83	1.86 ± 0.54	0.513			
Dissatisfaction for daily life subscale	2.43 ± 0.61	2.36 ± 0.80	2.07 ± 0.68	**0.062**	EG versus. GT	0.610	
					EG versus. DT	**0.012**	0.56
					GT versus. DT	**0.064**	0.38
Physical component summary*	49.39 ± 3.72	49.71 ± 3.29	50.47 ± 3.62	0.360			
Mental component summary*	48.24 ± 4.49	47.42 ± 5.42	48.14 ± 4.79	0.608			

In case the *P* value of ANOVA was less than 0.1, Tukey was conducted. When the *P* values were < 0.1 in Tukey, Cohen’s d was performed for the purpose of effect size. *p* < 0.05 was considered statistically significant. Cohen’s d means the effect of the variable of individual cause: values between 0.2 and < 0.5 denote a small but clinically meaningful difference between groups; values between 0.5 and < 0.8 denote a medium effect; and values ≥ 0.8 indicate a large effect. In items or subscales with*; higher score indicating better condition. In items or subscales without*; higher score indicating worse condition

## Discussion

The number of patients diagnosed with PGC is growing worldwide, especially those with early-stage PGC [2]. Radical resection and lymph node dissection are the Primary treatment options for GC. Various complications and dysfunctions occur in patients after gastrectomy, comprehensively called Postgastrectomy syndrome (PGS) [16]. PGS exhibits gastrointestinal symptoms associated with reduced gastric volume, considerably affecting patients' short-term postoperative recovery and long-term QOL [13]. Although the prime objective of gastrectomy is to treat tumours, it is also crucial to reduce PGS-related adverse effects to improve patients' postoperative QOL. This target is particularly relevant for patients with early PGC, as they need to face PGS for a long time [17].

Laparoscopic gastrectomy has become a common minimally invasive surgical procedure in recent years [18]. The appliance of laparoscopic techniques in PG gives EG more space for development and creativity. Therefore, PG has been gaining attention as a function-preserving procedure.

However, the high risk of PGS is headache of PG. As a classic reconstruction method after PG, esophagogastrostomy has the advantages of simple operation and minimal trauma. However, PG disrupts the normal anatomy of the esophagogastric junction and preserves the pyloric region leading to acid reflux and delayed gastric emptying. As a result, patients tend to have a higher incidence of PGS after PG. To reduce the prevalence of PGS and enhance patients' QOL, various methods have emerged to preserve the cardia's function or perform anti-reflux reconstruction. GT was first reported by Adachi et al. [19], which not only extends the distance of the reflux and reduces the gastric acid secretion to reduce the severity of reflux symptoms, but can reduce the anastomotic tension to ensure the anastomosis's safety [20]. Nevertheless, related research reported that anastomotic stenosis and reflux esophagitis remained high after GT. The postoperative complications of patients with GT have been reported in several studies [19, 21–25], and the rate of stenosis and reflux esophagitis was 7.1%-20%, and 5.7%-31.8%, respectively. In our studies, the outcome of postoperative complications was also unsatisfactory: the rate of anastomotic stenosis and reflux esophagitis was 5.2% and 23.4%.

Another viable reconstruction after PG is the Roux-en-Y type E-Jstomy [26]. One of the reconstruction modalities, DT, has been considered the most effective reconstructive procedure for anti-reflux [20]. Although DT requires three anastomoses (esophagojejunostomy, gastrojejunostomy, and jejunojejunostomy) and the procedures seem to be more complicated, there was no obvious difference in the anastomosis-related complications among the three groups. A study reported that no significant correlation between the number of anastomoses and the incidence of anastomotic leakage or stenosis [27]. Moreover, some retrospective studies [26, 28–32] reported the surgical outcomes after using DT for PG. Reflux esophagitis, reflux syndromes, and residual food were reported in 1.1–10.5%, 4.7–10.5%, and 0–48.9% of patients, respectively. In our series, outcomes in terms of postoperative complications in DT were superior to the other two groups.

Due to lack of food reserves and decreased appetite, patients often present with decreased food intake and weight loss after PG. In our study, we found that the EG group did not perform as well in terms of nutritional status. However, researchers had argued that although both EG and DT retained the same residual stomach volume, EG was considered to have a better nutritional condition [31]. The reason may be related to the high postoperative complications in the EG group. Complications such as reflux esophagitis and delayed gastric emptying lead to an insufficient quantity of diet and absorption of nutrients. In addition, Since DT provides two pathways for food transportation and storage, at least theoretically, its food storage capacity is not dependent on the volume of the residual stomach. Compared to the EG and DT group, the GT group did not have an advantage in short-term postoperative nutrition. This result was related to a relatively high rate of postoperative complications (reflux esophagitis, anastomotic stenosis), but more importantly, the decreased stomach volume after GT affected food intake and nutritional status [19].

We used the PGSAS-45, composed of questions on 45 items, which comprehensively evaluate the postgastrectomy symptoms, living status and QOL [13]. Several studies [14, 15, 33–35] have used the scale to assess QOL in patients after gastrectomy and demonstrated its validity, reliability and reproducibility. Among them, in some multicenter studies [15, 35], for GC patients, PG's superiority over TG in postoperative QOL was reported. However, few studies compared the QOL of several reconstruction techniques after PG.

In our study, differences were observed for the esophageal reflux subscale, change in body weight, dissatisfaction at the meal subscale, and dissatisfaction for daily life subscale among the main factors. The DT group was marginally better in the outcome of the esophageal reflux subscale. Furthermore, we found that the majority of patients only had mild symptoms. A study reported by Inada et al. [36] indicated that in EG after PG, favorable postoperative QOL may be associated with a larger residual stomach volume, anti-reflux procedures, less resection of the esophagus, the use of pylorostomy, and the preservation of the pyloric branch of the vagus nerve. Similar results in this study reflected that the GT group is not superior regarding the dissatisfaction at the meal subscale and dissatisfaction for daily life subscale. The reason for this outcome may be related to the fact that its reconstructive procedure requires the remnant stomach to be made into a tube, resulting in a smaller remnant stomach volume. As we expected, patients did not have an advantage in postoperative QOL in the EG group. This outcome is associated with a high rate of postoperative complications. Overall, the DT group had satisfactory results in terms of QOL.

However, there are some limitations in this study. First, this study is a retrospective study of a case series, and the sample size included in this study was relatively small; Second, we did not use clinical evaluations, anthropometric tests and laboratory tests to investigate overall functional results. We only evaluated the nutritional conditions by blood indicators and BMI; Third, only postoperative one-year data were shown in this study. Fourth, the effect of PPI (proton pump inhibitor) on gastric acid secretion could not be assessed because it was not possible to record the patient's PPI intake after discharge from the hospital. Fifth, this study did not clarify whether the size of the residual stomach affects the QOL and nutritional status of postoperative patients due to missing data of the remnant stomach size.

Notably, all three groups in the study had a higher incidence of reflux symptoms and reflux esophagitis. We considered that this may be related to the shortcomings of the present study. On the one hand, the sample size of this study is small, which may lead to biased outcomes. More crucially, since the study population consisted mostly of patients with early-stage gastric cancer, the postoperative review rates were low. This results in the majority of those with complete review records being symptomatic patients.

## Conclusion

This study compared the postoperative functional outcomes among the EG, GT and DT groups. In the EG group, although the patients had better nutritional status in the postoperative period, the incidence of reflux esophagitis was still the biggest problem, which also greatly reduced the patients' QOL. We performed GT (a modified anti-reflux reconstruction based on EG) to reducing the prevalence of reflux esophagitis; However, the rate of reflux symptoms and reflux esophagitis remained high in the GT group. In addition, the nutritional status results in the postoperative period were not satisfactory in the GT group. We found a significant advantage in the DT group regarding postoperative complications, nutritional status and QOL.

## Data Availability

The data presented in this study are available on reasonable request from the corresponding author, Dr. Jun Ren.

## Abbreviations

EG: Esophagogastrostomy
GT: Gastric tube
DT: Double-tract
LPG: Laparoscopic proximal gastrectomy
QOL: Quality of life
GC: Gastric cancer
PGC: Proximal gastric cancer
TG: Total gastrectomy
PG: Proximal gastrectomy
JGCG: Japanese Gastric Cancer Guidelines
DG: Distal gastrectomy
JIP: Jejunal interposition
JPI: Jejunal pouch interposition
E-Jstomy: Esophagojejunostomy
G-Jstomy: Gastrojejunostomy
J-Jstomy: Jejunojejunostomy
ASA: American Society of Anesthesiologists
BMI: Body Mass Index
LA: Los Angeles
TP: Total protein
ALB: Albumin
HB: Hemoglobin
PGSAS-45: Postgastrectomy Syndrome Assessment Scale-45
PGS: Postgastrectomy syndrome
OPG: Open proximal gastrectomy
GC: Gastric cancer
PPI: Proton pump inhibitor
XELOX: Capecitabine plus oxaliplatin
